# Particulate-steroid betamethasone added to ropivacaine in interscalene brachial plexus block for arthroscopic rotator cuff repair improves postoperative analgesia

**DOI:** 10.1186/s12871-016-0251-9

**Published:** 2016-10-04

**Authors:** Kunitaro Watanabe, Joho Tokumine, Tomoko Yorozu, Kumi Moriyama, Hideaki Sakamoto, Tetsuo Inoue

**Affiliations:** 1 Department of Anesthesiology, Kyorin University School of Medicine, 6-20-2 Sinkawa, Mitaka, Tokyo, 181-8611 Japan; 2 Department of Anesthesia, Hino Municipal Hospital, 4-3-1, Tamadaira, Hino, Tokyo, 191-0062 Japan

**Keywords:** Arthroscopic rotator cuff repair, Betamethasone, Interscalene brachial plexus block, Postoperative pain

## Abstract

**Background:**

Dexamethasone added to local anesthetic for brachial plexus block improves postoperative pain after arthroscopic rotator cuff repair, as compared with the use of local anesthetic alone. Dexamethasone is present in non-particulate form in local anesthetic solution, while betamethasone is partially present in particulate form. The particulate betamethasone gradually decays and is expected to cause its longer-lasting effect. This study investigated the postoperative analgesic effect of betamethasone added to ropivacaine for brachial plexus block in patients who underwent arthroscopic rotator cuff repair.

**Methods:**

This was a prospective, randomized, triple-blind study of 44 patients undergoing arthroscopic rotator cuff repair surgery. Ultrasound-guided interscalene brachial plexus block, involving 20 mL of 0.375 % ropivacaine (group R) or 19 mL of 0.375 % ropivacaine with 4 mg (1 mL) of betamethasone (group BR), was administered and surgery was performed under general anesthesia. After surgery, the pain score was recorded at 12 h after surgery, and on the first, second, and seventh postoperative day. Analgesia duration, offset time of motor block, frequency of rescue analgesic administration, postoperative nausea/vomiting, and sleep disturbance during the night after surgery were recorded. The numerical values were expressed as median [interquartile range]. *P* values < 0.05 were considered statistically significant.

**Results:**

The duration of analgesia was significantly prolonged in group BR (group BR: 19.1 h [16.6, 20.9 h], group R: 13.3 h [11.6, 16.5 h], *p* < 0.001). The pain scores at 12 h after surgery and on the first and seventh day after surgery were significantly lower in group BR than in group R. The duration of motor block was significantly prolonged in group BR. The frequency of rescue analgesic administration and the sleep disturbance rate were significantly lower in group BR. There was no difference in postoperative nausea/vomiting between the two groups.

**Conclusions:**

Betamethasone added to local anesthetic in interscalene brachial plexus block improved postoperative pain after arthroscopic rotator cuff repair, and betamethasone prolonged the duration of analgesia by almost 6 h.

**Trial registration:**

University Hospital Medical Information Network Center Clinical Trials Registration System (UMIN000012899).

**Electronic supplementary material:**

The online version of this article (doi:10.1186/s12871-016-0251-9) contains supplementary material, which is available to authorized users.

## Background

Pain control after arthroscopic rotator cuff repair is challenging for anesthesiologists [1], but interscalene brachial plexus block has been shown to offer effective pain relief after this procedure [1]. Continuous nerve block may be ideal for prolonging the analgesic effect, but it may cause unexpected events, such as catheter migration, leakage of anesthetics, and infection [2–6]. In most cases, arthroscopic shoulder surgery is performed as outpatient surgery, and management of ambulatory catheters is an important consideration in this context. On the other hand, a single injection of anesthetics offers analgesia for a limited duration.

Dexamethasone added to local anesthetic for brachial plexus block improves postoperative pain after arthroscopic rotator cuff repair, as compared with the use of local anesthetic alone [7–14].

In this study, we used betamethasone instead of the commonly used dexamethasone. Betamethasone is a long-acting corticosteroid, and is a stereoisomer of dexamethasone. Steroids can be classified as particulate and non-particulate, depending on their solubility and aggregation characteristics. Dexamethasone is present only in non-particulate form in saline, while betamethasone is present in both particulate and non-particulate form, depending on the composition of the solution [15]. Betamethasone is partially present in particulate form in ropivacaine solution. The particulate steroid is thought to act as a local reserve, which gradually decays and releases the steroid, thereby causing its longer-lasting effect.

Betamethasone has been primarily utilized for peripheral nerve injections in patients with chronic pain, because of its longer-lasting analgesia [16–19]. The conventional dose of perineural betamethasone is 2–8 mg. We chose 4 mg of betamethasone as a perineural adjuvant.

This study aimed to evaluate the analgesic effect of perineural betamethasone when added to ropivacaine in an interscalene brachial plexus block.

## Methods

This study was reviewed and approved by a local ethics committee (Hino Municipal Hospital Ethical Review Board; Reception. No. 25-11, approved on the 6 Mar 2014), and was registered in the University Hospital Medical Information Network Center Clinical Trials Registration System (UMIN000012899, registered on the 10 Mar 2014).

Informed consent was obtained from patients scheduled for arthroscopic rotator cuff repair from April 2014 to March 2015. Exclusion criteria were allergy against local anesthetics, coagulation disorder, local skin infection at the block site, peripheral neuropathy, pre-existing steroid administration, ASA physical status ≥ 4, and patient refusal. Patients were randomly divided in two groups, with (group BR) or without addition of betamethasone (group R) to ropivacaine for the interscalene plexus block.

A randomized, triple-blind selection of the groups was performed. Two nurses prepared the solution for the nerve block before the anesthesiologist entered the operation room. The solution for group BR was a combination of 9.5 mL of 0.75 % ropivacaine (Anapeine® 7.5 mg/mL, AstraZeneca, Osaka, Japan), 9.5 mL of saline, and 4 mg (1 mL) of betamethasone (Rinderon®, Shionogi Co., Osaka, Japan). Betamethasone was added after the 0.375 % ropivacaine solution was prepared, as steroid particles may precipitate when the ropivacaine stock solution and betamethasone are mixed. The solution for group R was a combination of 10 mL of 0.75 % ropivacaine and 10 mL of saline. The anesthesiologists, orthopedic surgeons, and nurses in the ward remained blinded to the patients’ group allocation until the end of the study.

General anesthesia was induced by administrating 2 mg/kg of propofol and 100–200 μg of fentanyl intravenously. Then, a laryngeal mask (ProSeal™, Teleflex, San Diego, CA, USA) was placed on the patient, and the anesthesia was maintained with 4–6 % desflurane. An ultrasound-guided interscalene plexus block was administered after placement of the laryngeal mask [20]. We used a 6–13 Hz high-frequency linear probe (HFL 38x EDGE, SonoSite Co., Bothell, WA, USA) for guiding the block. The anesthesiologist placed the probe at the level of C6 and identified the brachial plexus running between the anterior and median scalene muscles. A 22G needle (Stimplex® Ultra, B. Braun, Melsungen, Germany) was used to administer the nerve block. Although the brachial plexus was mostly identified only by ultrasound, we used ultrasound and nerve stimulation if there was any difficulty in identifying the brachial plexus [21, 22]. When the needle had been placed at the nerves between C5 and C6, 20 mL of the prepared solution was injected slowly. All nerve blocks were performed by anesthesiologists who were experts in administration of ultrasound-guided nerve blocks. During surgery, ephedrine and/or phenylephrine were administered to maintain appropriate hemodynamics in the patients. At the end of the surgery, 50 mg of flurbiprofen was routinely administered intravenously. We verified whether the nerve block was sufficient, by a pain score of zero, sensory loss, and motor block after the patient’s awakening from general anesthesia.

The primary outcome of this study was the duration of analgesia, which was assessed by the time to first analgesic request. Secondary outcome measures included offset time for motor block, consumption of rescue analgesics, and the presence of sleeping disturbance during the night after the operation day were recorded (See: Additional file 1).

Postoperative pain was scored on the Wong-Baker Face Scale (scale range: 0–5; 0: no pain, 5: strongest pain) on the day of surgery (12 h after surgery), as well as on the first, second, and seventh postoperative day (POD). Motor block was assessed on the modified Lovett rating scale (0: complete paralysis, 1: almost complete paralysis, 2: pronounced mobility impairment, 3: slight mobility impairment, 4: pronounced reduction of muscular force, 5: slightly reduced muscular force, 6: normal muscular force). Offset time for motor block was measured as the time lapsed till returning to baseline muscular force of thumb abduction (radial nerve), thumb adduction (ulnar nerve), thumb opposition (median nerve), and elbow flexion (musculocutaneous nerve) [23]. The postoperative pain score and the motor block rating scale were measured by nurses attending the orthopedics ward.

The nurses were educated about stating the pain scores and the neurologic evaluation prior to study commencement. Postoperatively, the patients were allowed to request rescue analgesics at any time, and received diclofenac (25 mg p.r.) with at least a 6-h interval before re-administration. If the diclofenac did not relieve the pain, patients received pentazocine (15 mg i.v.) with an interval of at least 30 min. On the first postoperative day, the regular administration of 4 mg of oral lornoxicam after each meal was started and continued until the fifth postoperative day. Administration times and the amount of analgesics used were recorded by the ward nurses.

Systemic or surgical site infection was evaluated by two observers, an orthopedic surgeon not in charge of the patient, and an infection control nurse in the hospital.

Sample size was calculated from the data of time to first analgesic request in a pilot study. The sample size required for 80 % power at ɑ = 0.05 was estimated to be 22 patients each for the experimental and control group. We used the Wilcoxon test to compare continuous variables and Fisher’s exact test to compare nominal variables. The numerical values were expressed as ratios (%) or as the median (interquartile range). *P* values less than 0.05 were considered statistically significant. We performed the log-rank test to compare the duration of the analgesic effect. Statistical analyses were performed with JMP 11 statistical software (JMP Statistical Discovery, Cary, NC, USA).

## Results

Forty-four patients participated in this study. No patient was excluded using our criteria. All patients were healthy (ASA physical status 1 or 2), and there was no difference in the demographic data among the groups, except for the sex ratio (Table 1). We performed a subgroup analysis for each sex, but found no statistically significant difference in the primary outcome measure.

**Table 1:** Demographic data

Variable	Group R (*n* = 22)	Group BR (*n* = 22)	*p* value
Age (years)	65 (58,69)	65 (60,70)	0.25
Sex male/female	15/7	7/15	0.034*
ASA status 1/2	10/12	12/10	0.76
BMI	25 (23,27)	23 (19,25)	0.06
Right/Left	8/14	8/14	1.00
Anchors	3 (3,4)	3 (1.75,4)	0.25
Surgical time (min)	108 (90,133)	95 (74,115)	0.11
Fentanyl (mcg)	100 (100,163)	100 (100,150)	0.77

There was no difference in the demographic data, except for the sex ratio

All measured values are presented as median (interquartile range) or numbers of patients. Group R: 0.375 % ropivacaine (20 mL), group BR: betamethasone 4 mg (1 mL) + 0.375 % ropivacaine (19 mL), ASA: American Society of Anesthesiologist, BMI: Body mass index, Anchors: number of implanted anchors. *P < 0.05. (See: Additional file 1)

There was no technical difficulty in identifying the brachial plexus with ultrasound devices and the nerve block was performed sufficiently in all cases. There were no neurological complications or infections (systemic or surgical site).

**Fig. 1:**
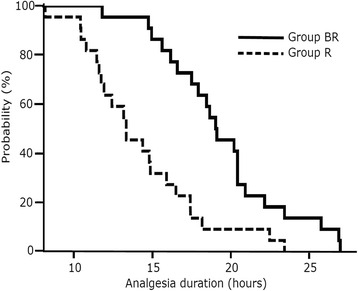
Duration of analgesia. The duration of analgesia in group BR (solid line) was significantly longer than that in group R (*dotted line*). R group: 0.375 % ropivacaine (20 mL), BR group: betamethasone 4 mg (1 mL) + 0.375 % ropivacaine (19 mL)

The duration of analgesia in group BR was significantly longer than that in group R (group BR: 19.1 h [16.6, 20.9 h], group R: 13.3 h [11.6, 16.5 h], *p* < 0.001; Fig. 1).

**Fig. 2:**
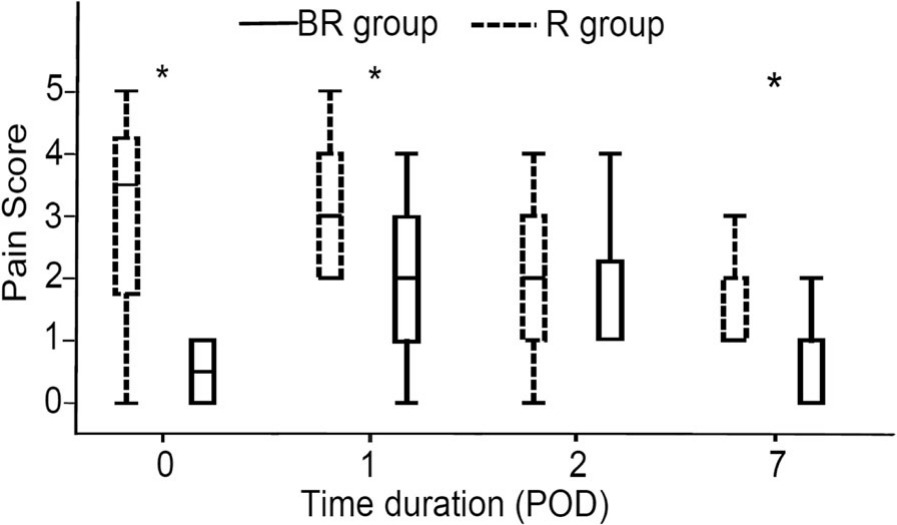
Postoperative pain scores. The pain score (Wong − Baker Face Scale) of group BR (solid line) was significantly lower than that of group R (dotted line) during the night after the operation, and on the first and seventh postoperative day, but not on the second postoperative day. POD: postoperative day, R group: 0.375 % ropivacaine (20 mL), BR group: betamethasone 4 mg (1 mL) + 0.375 % ropivacaine (19 mL). *: *p* < 0.05

The pain score in group BR was significantly lower than that in group R at 12 h after surgery (group BR: 0.5 [0, 1], group R: 3.5 [1.75, 4.25], *p* < 0.001), on POD 1 (group BR: 2.0 [1, 2], group R: 3.0 [2, 4], *p* = 0.005) and POD 7 (group BR: 1.0 [0, 1], group R: 2.0 [1, 2], *p* < 0.001), but not on POD 2 (group BR: 1 [1, 2.25], group R 2[1, 3], *p* = 0.18; Fig. 2).

The frequency of sleep disturbance during the night after surgery in group BR was significantly lower than that in group R (group BR: 14 %, group R: 77 %, *p* < 0.001). The amount of rescue analgesics in group BR was significantly lower than that in group R (Table 2). The frequency of nausea/vomiting in the groups was not significantly different (Table 2).

**Table 2:** Presence of sleep disturbance and the consumption of analgesics after surgery

Variable	Group R (*n* = 22)	Group BR (*n* = 22)	*P* value
Sleep disturbance	17 (77 %)	3 (14 %)	<0.001
Rescue analgesics			
Diclofenac (mg)	50 (18.5, 50)	0 (0. 6.3)	<0.001
Pentazocine(mg)	0 (0, 15)	0 (0, 0)	0.004
Nausea/ vomiting (%)	0 (0 %)	2 (9 %)	0.490

Values represent the number of patients (percentage) with sleep disturbance and nausea/vomiting. Consumption of rescue analgesics (mg) is expressed as median (interquartile range)

R group: 0.375 % ropivacaine (20 mL), BR group: betamethasone 4 mg (1 mL) + 0.375 % ropivacaine (19 mL)

The duration of the motor block in group BR was significantly longer than that in group R (group BR: 13.9 h [8.2, 18.1 h], group R: 10.4 h [3.9, 13.2 h], *p* = 0.004).

## Discussion

Our study showed that perineural betamethasone added to ropivacaine prolonged the duration of analgesia by almost 6 h. This is the first report to show a long-term effect of particulate steroid when used as an anesthetic adjuvant. Kawanishi et al. reported that dexamethasone added to 0.75 % ropivacaine prolonged the duration of analgesia by almost 7 h [14]. Our result also showed prolonged analgesia duration using 0.375 % ropivacaine, which was half of the concentration of ropivacaine used in the paper by Kawanishi et al. [14].

Although the analgesia-prolonging effect of perineural betamethasone was statistically significant at postoperative day 7, the effect was slight. Therefore, the long-term effect in terms of clinical efficacy remains unclear.

This was a randomized control study. We planned the sample size used from the data obtained from a pilot study. However, the sex distribution was statistically significantly different between the two groups. In the post-hoc statistical analysis based on gender, no significant differences in primary outcome were obtained.

Infarction of the spinal cord due to using betamethasone for epidural analgesia has previously been reported [24]. This was thought to be due to large particles of betamethasone occluding the blood vessels supplying the spinal cord. On the other hand, betamethasone has been used clinically for nerve root blocks [25]. We therefore speculate that perineural injection of betamethasone is safe.

Steroids also have anti-emetic effects [26, 27], but we could not show this effect in the present study. It is possible that the amount of betamethasone used was too little to exert an antiemetic effect. We surmise that betamethasone stayed at the injected site, and that the systemic blood concentration may not have attained sufficient levels to exert an anti-emetic effect.

This study had some limitations. We did not directly compare betamethasone with dexamethasone. The difference in analgesic effect between betamethasone and dexamethasone remains unknown. We chose to use 4 mg of betamethasone for this study. This dose was arbitrarily chosen, given that the conventional dose of betamethasone ranges from 2 to 8 mg. We did not perform a proper dose-ranging study with betamethasone as a local anesthetic adjuvant for an interscalene brachial plexus block. Further studies are needed to elucidate the long-term effect and optimal dose of particulate betamethasone.

## Conclusions

Betamethasone added to ropivacaine in interscalene brachial plexus block improves postoperative pain after arthroscopic rotator cuff repair, and betamethasone prolonged the duration of analgesia by almost 6 h.

## Additional file

Additional file 1: This is raw data of our clinical research. This file includes all our data of demographic data, primary outcome and secondary outcome. (XLS 33 kb)

## Abbreviations

ASA: American Society of Anesthesiologist
BMI: Body mass index
POD: Postoperative day
